# Assessment of 90-Day Outcomes Following Total Joint Arthroplasty in Ambulatory Surgery Centers, Hospital Outpatient Departments, and Hospitals: A Michigan Arthroplasty Registry Collaborative Quality Initiative Analysis

**DOI:** 10.1016/j.artd.2025.101659

**Published:** 2025-03-08

**Authors:** Simarjeet Puri, Martin Weaver, Lisheng Chen, Tae Kim, Elizabeth Dailey, David C. Markel

**Affiliations:** a Section of Orthopedic Surgery, Ascension Providence Hospital, Southfield, MI, USA; b Michigan Arthroplasty Registry Collaborative Quality Initiative, Ann Arbor, MI, USA; c Department of Surgery and Biomedical Engineering, University of Michigan, Ann Arbor, MI, USA; d Department of Orthopedic Surgery, University of Michigan, Ann Arbor, MI, USA; e The Core Institute, Novi, MI, USA

**Keywords:** Registry study, Outpatient surgery, Total joint arthroplasty

## Abstract

**Background:**

Total joint arthroplasty is shifting from hospitals to ambulatory surgery centers (ASCs) and hospital outpatient departments (HOPDs). A Michigan Arthroplasty Registry Quality Collaborative Initiative quality improvement project examined readmissions, emergency room (ER) visits, periprosthetic joint infection (PJI), fracture, and dislocation after primary total hip arthroplasty (THA) or total knee arthroplasty (TKA) across sites.

**Methods:**

Primary TJAs between July 1, 2021, and June 30, 2022 (N = 41,696: 3910 ASC, 1,834 HOPD, and 35,952 hospital) were reviewed. Of 17,100 THAs, 9.5% (1,631) were at ASCs, 4.7% (798) at HOPDs, and 85.8% (14,671) at hospitals. Of 24,596 TKAs, 9.3% (2,279) were at ASC, 4.2% (1,036) at HOPDs, and 86.5% (21,281) at hospitals. Hospitals treated more elderly, women, non-White, obese, diabetics, smokers, and governmental insurance.

**Results:**

For THAs, ASCs had the lowest 30-day (ASC 1%, HOPD 1.8%, hospital 3.4%, *P* < .001) and 90-day (ASC 1.7%, HOPD 3.4%, hospital 5.5%, *P* < .001) readmissions, 30-day ER visits (ASC 1.8%, HOPD 3.5%, hospital 5.3%, *P* < .001), and fractures (ASC 0.4%, HOPD 0.6%, hospital 1.2%, *P* < .001). Similar trends were observed for TKAs: 30-day readmissions (ASC 1.3%, HOPD 1.4%, hospital 3.1%, *P* < .001), 90-day readmissions (ASC 2.2%, HOPD 2.3%, hospital 5.2%, *P* < .001), and 30-day ER visits (ASC 3%, HOPD 6.5%, hospital 6.4%, *P* < .001). PJI (THA: *P* = .1, TKA: *P* = .6) and dislocation rates (*P* = .5) were similar across sites.

**Conclusions:**

Patients receiving primary total joint arthroplasty at an ASC had the least postoperative hospital-based care despite similar rates of PJI and dislocation.

## Introduction

Two million primary total hip arthroplasty (THA) and total knee arthroplasty (TKA) are performed annually in the United States, with demand expected to double by 2050 [1]. Advances in surgical techniques, perioperative protocols, and multimodal analgesia regimens have reduced hospital stays for THA (1.4 days) and TKA (1.3 days) [2]. In response, the Centers for Medicaid and Medicare Services removed THA and TKA from its inpatient-only list and added them to the Ambulatory Surgery Center Payable list [3,4]. With Medicare joining commercial insurers in covering primary total joint arthroplasty (TJA) at ambulatory surgery centers (ASCs) and hospital outpatient departments (HOPDs), site of service has shifted significantly. Projections suggest that by 2026, half of all TJAs will be performed in outpatient settings [5,6].

The COVID-19 pandemic further accelerated this since many hospitals suspended elective surgeries, making ASCs and HOPDs the only options for elective TJA [4]. A previous Michigan Arthroplasty Registry Quality Collaborative Initiative (MARCQI) study reported an 84% and 125% increase in ASC/HOPD THAs and TKAs, respectively, during this period [7]. Compared to prepandemic, patients with more comorbidities and older age began having primary TJA in ASCs and HOPDs but continued to demonstrate lower readmissions and similar rates of emergency room (ER) visits, periprosthetic joint infection (PJI), periprosthetic hip fracture, and prosthetic hip dislocation compared to TJA performed in hospitals [7].

The purpose of this follow-up study was to describe health-care trends in all-cause 30-day and 90-day readmissions, all-cause 30-day ER visits, PJI, periprosthetic hip fracture, and prosthetic hip dislocation after primary TJA at Michigan’s ASCs, HOPDs, and hospitals after the COVID-19 pandemic. Whereas many of the studies investigating outcomes after inpatient vs outpatient TJA have utilized claims-based datasets, [5,[8], [9], [10]] which rely on accurate coding of complications or single-institution records [11,12]. MARCQI is the largest statewide database in the United States and includes more than 500,000 fully abstracted cases and captures data on 96% of Michigan TJAs [7]. We hypothesized broader utilization of ASCs compared to prepandemic trends as well as continued low rates of readmission, ER visits, and complications among ASC TJAs when compared to HOPDs and hospitals.

## Material and methods

**Figure 1:**
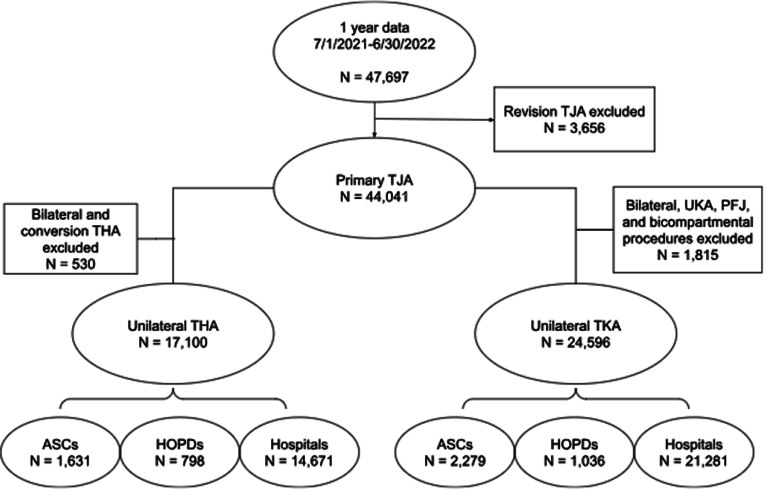
Data flow chart describing inclusion and exclusion criteria used in the study. UKA, unicondylar knee arthroplasty; PFJ, patellofemoral joint arthroplasty.

The MARCQI database carries the determination of “not-regulated” under Title 45, Part 46 of the Code of Federal Regulations by the University of Michigan’s Institutional Review Board. Data are specifically abstracted by trained nurses at each participating site. Within 90 days of surgery, charts are prospectively reviewed and events are captured by trained clinical abstractors. Beyond that time, a prospectively captured statewide database is utilized to confirm readmissions, even outside the operative site [13]. Using the MARCQI database, all primary, unilateral THAs and TKAs were reviewed from July 1, 2021, through July 30, 2022, corresponding with 1 year after the COVID-19 elective surgery shutdown. Conversion THA, unicompartmental knee arthroplasty, bicompartmental knee arthroplasty, and patellofemoral arthroplasty were excluded (Fig. 1). Demographic data including age, sex, body mass index (BMI), race, diabetes status, smoking status, American Society of Anesthesiologists (ASA) classification, and insurance type (government vs nongovernment) were collected. All-cause readmissions and ER visits within 30 and 90 days were identified. PJI, defined as per Parvizi [14], periprosthetic hip fracture, and prosthetic hip dislocation were noted. The study group included a total of 41,696 primary TJAs performed at 65 hospitals, 6 HOPDs, and 11 surgery centers across the state. The cohorts were evaluated for demographics including age, sex, BMI, diabetes status, ASA classification, smoking status, insurance type, and race for both primary THA (Table 1) and TKA (Table 2).

**Table 1:** Demographics of primary THA performed at ambulatory surgery centers (ASCs), hospital outpatient departments (HOPDs), and hospitals.

Variable	Overall	Surgery location			*P* values
		ASC	HOPD	Hospital	
Continuous variable	Mean (±SD)	Mean (±SD)	Mean (±SD)	Mean (±SD)	
Age (y)	66 (±10.5)	62.9 (±9.4)	62.7 (±9.7)	66.5 (±10.6)	<.01
BMI (kg/m2)	30.7 (±6.3)	29.5 (±5.3)	30.2 (±5.1)	30.9 (±6.4)	<.01

Categorical variables	N (%)	N (%)	N (%)	N (%)	
BMI (reclassification)					<.01
Underweight	110 (0.6%)	9 (0.6%)	0 (0.0%)	101 (0.7%)	
Normal	2,870 (16.8%)	318 (19.5%)	110 (13.8%)	2,442 (16.7%)	
Preobese	5,479 (32.0%)	606 (37.2%)	303 (38.0%)	4,570 (31.2%)	
Obese class I	4,623 (27.0%)	439 (26.9%)	235 (29.5%)	3,949 (26.9%)	
Obese class II	2,742 (16.0%)	209 (12.8%)	124 (15.5%)	2,409 (16.4%)	
Obese class III	1,276 (7.5%)	50 (3.1%)	26 (3.3%)	1,200 (8.2%)	
Sex					<.01
Female	9,330 (54.6%)	801 (49.1%)	429 (53.8%)	8,100 (55.2%)	
Male	7,760 (45.4%)	828 (50.8%)	369 (46.2%)	6,563 (44.7%)	
Unknown	10 (0.1%)	2 (0.1%)	0 (0.0%)	8 (0.1%)	
Diabetes					<.01
Nondiabetic	14,298 (83.6%)	1,493 (91.5%)	701 (87.8%)	12,104 (82.5%)	
Diabetic	2,799 (16.4%)	138 (8.5%)	97 (12.2%)	2,564 (17.5%)	
Unknown	3 (0.0%)	0 (0.0%)	0 (0.0%)	3 (0.0%)	
ASA					<.01
I	359 (2.1%)	110 (6.7%)	38 (4.8%)	211 (1.4%)	
II	8,463 (49.5%)	1,241 (76.1%)	527 (66.0%)	6,695 (45.6%)	
III	7,994 (46.8%)	272 (16.7%)	233 (29.2%)	7,489 (51.1%)	
IV	272 (1.6%)	0 (0.0%)	0 (0.0%)	272 (1.9%)	
Unknown	12 (0.1%)	8 (0.5%)	0 (0.0%)	4 (0.0%)	
Smoking status					<.01
Never	8,620 (50.4%)	1,005 (61.6%)	446 (55.9%)	7,169 (48.9%)	
Previous	6,130 (35.9%)	463 (28.4%)	277 (34.7%)	5,390 (36.7%)	
Current	2,298 (13.4%)	160 (9.8%)	75 (9.4%)	2,063 (14.1%)	
Unknown	52 (0.3%)	3 (0.2%)	0 (0.0%)	49 (0.3%)	
Insurance					<.01
Government	9,855 (57.6%)	636 (39.0%)	322 (40.4%)	8,897 (60.6%)	
Nongovernment	7,245 (42.4%)	995 (61.0%)	476 (59.7%)	5,774 (39.4%)	
Race					<.01
Caucasian	14,633 (85.6%)	1,271 (77.9%)	679 (85.1%)	12,683 (86.5%)	
Black	1,489 (8.7%)	58 (3.6%)	93 (11.7%)	1,338 (9.1%)	
Asian	40 (0.2%)	3 (0.2%)	3 (0.4%)	34 (0.2%)	
Native American	38 (0.2%)	2 (0.1%)	1 (0.1%)	35 (0.2%)	
Native Hawaiian-Pacific Islander	1 (0.0%)	0 (0.0%)	0 (0.0%)	1 (0.0%)	
Other/unknown	899 (5.3%)	297 (18.2%)	22 (2.8%)	580 (4.0%)

**Table 2:** Demographics of primary TKA performed at ambulatory surgery centers (ASCs), hospital outpatient departments (HOPDs), and hospitals.

Variable	Overall	Surgery location			*P* values
		ASC	HOPD	Hospital	
Continuous variable	Mean (±SD)	Mean (±SD)	Mean (±SD)	Mean (±SD)	
Age (y)	67.4 (±9.2)	64.9 (±8.1)	65.6 (±8.2)	67.7 (±9.3)	<.01
BMI (kg/m2)	33.0 (±6.6)	31.6 (±5.7)	32.0 (±5.6)	33.2 (±6.7)	<.01

Categorical variables	N (%)	N (%)	N (%)	N (%)	
BMI (reclassification)					<.01
Underweight	55 (0.2%)	6 (0.3%)	2 (0.2%)	47 (0.2%)	
Normal	2,275 (9.3%)	256 (11.2%)	101 (9.8%)	1,918 (9.0%)	
Preobese	6,490 (26.4%)	716 (31.4%)	297 (28.7%)	5,477 (25.7%)	
Obese class I	7,164 (29.1%)	708 (31.1%)	330 (31.9%)	6,126 (28.8%)	
Obese class II	5,123 (20.8%)	417 (18.3%)	225 (21.7%)	4,481 (21.1%)	
Obese class III	3,489 (14.2%)	176 (7.7%)	81 (7.8%)	3,232 (15.2%)	
Sex					<.01
Female	15,050 (61.2%)	1,216 (53.4%)	629 (60.7%)	13,205 (62.1%)	
Male	9,544 (38.8%)	1,063 (46.6%)	407 (39.3%)	8,074 (37.9%)	
Unknown	2 (0.0%)	0 (0.0%)	0 (0.0%)	2 (0.0%)	
Diabetes					<.01
Nondiabetic	19,165 (77.9%)	1,966 (86.3%)	858 (82.8%)	16,341 (76.8%)	
Diabetic	5,425 (22.1%)	313 (13.7%)	178 (17.2%)	4,934 (23.2%)	
Unknown	6 (0.0%)	0 (0.0%)	0 (0.0%)	6 (0.0%)	
ASA					<.01
I	256 (1.0%)	93 (4.1%)	10 (1.0%)	153 (0.7%)	
II	10,699 (43.5%)	1,681 (73.8%)	611 (59.0%)	8,407 (39.5%)	
III	13,294 (54.1%)	501 (22.0%)	415 (40.1%)	12,378 (58.2%)	
IV	340 (1.4%)	1 (0.0%)	0 (0.0%)	339 (1.6%)	
Unknown	7 (0.0%)	3 (0.1%)	0 (0.0%)	4 (0.0%)	
Smoking status					<.01
Never	13,659 (55.5%)	1,418 (62.2%)	596 (57.5%)	11,645 (54.7%)	
Previous	8,753 (35.6%)	698 (30.6%)	377 (36.4%)	7,678 (36.1%)	
Current	2,083 (8.5%)	158 (6.9%)	62 (6.0%)	1,863 (8.8%)	
Unknown	101 (0.4%)	5 (0.2%)	1 (0.1%)	95 (0.5%)	
Insurance					<.01
Government	14,949 (60.8%)	1,033 (45.3%)	533 (51.5%)	13,383 (62.9%)	
Nongovernment	9,647 (39.2%)	1,246 (54.7%)	503 (48.6%)	7,898 (37.1%)	
Race					<.01
Caucasian	20,496 (83.3%)	1,782 (78.2%)	799 (77.1%)	17,915 (84.2%)	
Black	2,400 (9.8%)	111 (4.9%)	182 (17.6%)	2,107 (9.9%)	
Asian	210 (0.9%)	6 (0.3%)	16 (1.5%)	188 (0.9%)	
Native American	68 (0.3%)	8 (0.4%)	3 (0.3%)	57 (0.3%)	
Native Hawaiian-Pacific Islander	20 (0.1%)	4 (0.2%)	1 (0.1%)	15 (0.1%)	
Other/unknown	1,402 (5.7%)	368 (16.2%)	35 (3.4%)	999 (4.7%)	

The Pearson chi-squared tests were used to compare categorical variables. Further pairwise comparisons between ASC vs hospital, ASC vs HOPD, and HOPD vs hospital were made without adjustment. Analysis of variance tests were used to compare continuous variables across the 3 cohorts. An alpha level of 0.05 was used to determine statistical significance. All tests were two-tailed. All analyses were performed using SAS 9.4 TS level 1M4.

Of note, support for the MARCQI is provided by Blue Cross Blue Shield of Michigan (BCBSM) and Blue Care Network as part of the BCBSM Value Partnerships program. Although Blue Cross Blue Shield of Michigan and the MARCQI work collaboratively, the opinions, beliefs, and viewpoints expressed by the author do not necessarily reflect the opinions, beliefs, and viewpoints of BCBSM or any of its employees.

## Results

There were 41,696 primary TJAs included in the study, of which 3,910 were at ASC, 1,834 at HOPD, and 35,952 at the hospital. There were 17,100 THAs: 9.5% (1,631) were performed in ASCs, 4.7% (798) at HOPDs, and 85.8% (14,671) at hospitals. There were 24,596 TKAs: 9.3% (2,279) were performed in ASC, 4.2% (1,036) in HOPDs, and 86.5% (21,281) in hospitals. The 3 cohorts were significantly different in terms of age, sex, BMI, diabetes status, ASA classification, smoking status, insurance type, and race for both primary THA (Table 1) and TKA (Table 2). The hospital population included higher percentages of elderly, women, non-White population, obesity, American Society of Anesthesiology class 3 or 4, diabetes, current smokers, and governmental insurance compared to ASCs or HOPDs. We further evaluated the length of stay for the hospital cohort. After primary THA, 31.1% (4,564) were discharged the same day, 50.2% (7,362) stayed 1 night, and 18.7% (2,745) stayed more than 1 night. Similarly, after primary TKA, 32.6% (6,928) were discharged the same day, 50.7% (10,781) stayed 1 night, and 16.8% (3,572) stayed more than 1 night.

### Readmissions

Primary THAs performed at ASCs had the lowest rate of 30-day and 90-day readmissions than HOPDs, and hospitals had the highest (Table 3). In pairwise analyses, 30-day readmissions were significantly lower in ASCs vs hospitals (1% vs 3.4%, *P* < .001) and HOPDs vs hospitals (1.8% vs 3.4%, *P* = .01), but were not different between ASCs and HOPDs (1% vs 1.8%, *P* = .1) (Table 4). For 90-day readmissions, rates were significantly lower in ASCs vs hospitals (1.7% vs 5.5%, *P* < .001), ASC vs HOPDs (1.7% vs 3.4%, *P* = .01), and HOPDs vs hospitals (3.4% vs 5.5%, *P* = .01) (Table 4).

**Table 3:** Readmissions and complications after primary THA across ASCs, HOPDs, and hospitals.

Outcome	ASC (n = 1,631)	HOPD (n = 798)	Hospital (n = 14,671)	*P* Value
30-d readmission	1%	1.8%	3.4%	<.001
90-d readmission	1.7%	3.4%	5.5%	<.001
30-d ER visits	1.8%	3.5%	5.3%	<.001
PJI	0.3%	0.4%	0.6%	.1
THA fracture	0.4%	0.6%	1.2%	.001
THA dislocation	0.5%	0.8%	0.7%	.5

P value determined by chi-square test.

**Table 4:** Pairwise *P* values comparing primary THA across ASCs, HOPDs, and hospitals.

Outcome	ASC vs hospital	ASC vs HOPD	HOPD vs hospital
30-d readmission	**<0.001**	0.1	**0.01**
90-d readmission	**<0.001**	**0.01**	**0.01**
30-d ER visits	**<0.001**	**0.01**	**0.03**
PJI	0.07	0.6	0.4
THA fracture	**0.002**	0.4	0.1
THA dislocation	0.2	0.3	0.8

Bold values indicate significance of 0.05.

Primary TKAs performed at ASCs had the lowest rates of 30-day and 90-day readmissions than HOPDs, followed by hospitals (Table 5). In pairwise analyses, 30-day readmissions were significantly lower for ASCs vs hospitals (1.3% vs 3.1%, *P* < .001) and HOPDs vs hospitals (1.4% vs 3.1%, *P* < .001), but again not between ASCs and HOPDs (1.3% vs 1.4%, *P* = .9) (Table 6). Similar patterns were seen for 90-day readmissions comparing ASCs vs hospitals (2.2% vs 5.1%, *P* < .001), HOPDs vs hospitals (2.3% vs 5.1%, *P* < .001), and ASC vs HOPDs (2.2% vs 2.3%, *P* = .8) (Table 6).

**Table 5:** Readmissions and complications after primary TKA across ASCs, HOPDs, and hospitals.

Outcome	ASC (n = 2,617)	HOPD (n = 1,110)	Hospital (n = 22,619)	*P* value
30-day readmission	1.3%	1.4%	3.1%	<.001
90-day readmission	2.2%	2.3%	5.1%	<.001
30-day ER visits	3%	6.5%	6.4%	<.001
PJI	0.3%	0.3%	0.4%	.6

P value determined by chi-square test.

**Table 6:** Pairwise *P* values comparing primary TKA across ASCs, HOPDs, and hospitals.

Outcome	ASC vs hospital	ASC vs HOPD	HOPD vs hospital
30-day readmission	**<0.001**	0.9	**0.002**
90-day readmission	**<0.001**	0.8	**<0.001**
30-day ER visits	**<0.001**	**<0.001**	0.9
PJI	0.4	0.9	0.6

Bold values indicate significance of 0.05.

### Emergency room visits

For the rate of ER visits, primary THA performed at ASCs was the lowest, followed by HOPDs, and then hospitals (Table 3). In pairwise analyses, 30-day ER visits were significantly lower in comparing ASCs vs hospitals (1.8% vs 5.3%, *P* < .001), HOPDs vs hospitals (3.5% vs 5.3%, *P* = .03), and ASCs vs HOPDs (1.8% vs 3.5%, *P* = .01) (Table 4). For TKA, those performed at ASCs were the lowest, followed by hospitals, and now HOPDs had the highest rate of ER visits (Table 5). In pairwise analyses, 30-day ER visits were significantly lower in ASCs vs hospitals (3% vs 6.4%, *P* < .001) and ASCs vs HOPDs (3% vs 6.5%, *P* < .001), but not HOPDs vs hospitals (6.5% vs 6.4%, *P* = .9) (Table 4).

### Complications

For both primary THA (Table 3) and TKA (Table 5), the rate of PJI was not significantly different among sites of service. Pairwise analyses did not reveal any significant differences between ASC, HOPD, or hospitals (Table 4, Table 6). Rates of periprosthetic hip fracture after primary THA were lowest at ASCs, then HOPDs, and highest at hospitals (Table 3). Pairwise analyses showed significantly lower periprosthetic hip fracture in ASC vs hospitals (0.4% vs 1.2%, *P* = .002), but not in ASC vs HOPDs (0.4% vs 0.6%, *P* = .4) or HOPDs vs hospitals (0.6% vs 1.2%, *P* = .1) (Table 4). Lastly, dislocation rates were similar across all 3 cohorts (Table 3, Table 4).

## Discussion

To our knowledge, this is the first study directly studying trends in readmission, ER visits, and complications after TJA across ASCs, HOPDs, and hospitals after the COVID-19 pandemic. Our findings contribute to a growing body of evidence that patient selection for TJA at standalone surgical centers continues to broaden, with increasing numbers of higher-risk patients being treated in these settings compared to prepandemic trends [7,[15], [16], [17], [18], [19]]. Despite this shift, readmissions and ER visits remain low, suggesting that surgeons are effectively selecting appropriate candidates for ASCs and HOPDs [5,[20], [21], [22], [23], [24]]. Our regional patient population, encompassing multiple institutions and practice types, validates this trend in an abstracted dataset. ASC patients not only had the lowest rates of readmission and 30-day all-cause ER visits but also were the highest percentage of nonsmokers, ASA class 1-2 patients, and nondiabetics receiving primary TJA. In contrast, hospitals continue to bear the burden of caring for the sickest patients undergoing primary TJA, and, not surprisingly, had the highest readmissions, ER visits, and complications.

Compared to HOPDs, ASCs demonstrated significantly lower ER visits for both primary THA and TKA as well as lower readmission rates for primary THA. These results differ from Wilson et al [25], who reported similar 90-day ER visits and readmissions between ASC and HOPD groups after primary TKA. This discrepancy may be explained by higher percentages of females, Black population, government insurance, ASA 3, and diabetic patients in the HOPD cohort. Notably, the study by Wilson et al [25] had similar BMI and percentage of females across HOPD and ASC cohorts but did not provide detailed demographic data beyond this. Both ASC and HOPD patients are selected for their ability to discharge home on the day of surgery [25]. They are typically younger, healthier, and with better support networks compared to patients treated in the hospital [25]. While prior studies have shown increased patient satisfaction in ASCs [26,27]. HOPDs, which are typically located adjacent to hospitals, can be advantageous for managing unforeseen complications after surgery [25]. This may explain the demographic differences seen in the HOPD cohort as well as the high number of 30-day ER visits among HOPD TKA observed in our study.

During the COVID-19 pandemic, inpatient elective procedures were postponed for resource preservation and incentivized a site of service change to the outpatient setting [6,7]. A previous MARCQI publication by Powell et al described an 84% increase in THAs and a 125% increase in TKAs performed at ASCs and HOPDs; patients that were older and with higher ASA classification were more likely to be treated at ASCs and HOPDs as compared to before the pandemic [7].The study also described lower readmission but similar ER visits and complications among TJAs performed at ASCs and HOPDs to those performed in the hospital [7].Some criticisms of this study were that readmissions, ER visits, and complications may have been falsely low in the setting of the COVID-19 pandemic when patients may have been hesitant to come to the hospital with postoperative complaints. However, this study corroborates the trends described by Powell et al [7] with further evidence showing that ASC patients in 2021 and 2022 were older, had higher BMI, a higher percentage of diabetics, and ASA class 3-4 compared to those in 2020. Two years after the pandemic in Michigan, this follow-up study described continued expansion of the ASC patient population with favorable outcomes relative to HOPDs and hospitals.

Another notable finding is the increased number of THA fractures in the hospital group, which had doubled the number of fractures compared to the HOPD cohort and triple that of the ASC group. Periprosthetic femur fractures are a common cause of early revision, especially in high-risk populations [28]. There are many risk factors that can increase the risk of periprosthetic femur fracture after THA, including female sex, old age, number of comorbidities, cementless fixation, collarless implants, and anterior approach [29,30]. A previous MARCQI study also determined high-volume surgeons, defined as greater than 404 THAs per year, were less likely to have postoperative femur fracture [31]. While these differences may be attributable to a more elderly, medically complex, and female hospital population, other postoperative complications such as PJI and THA dislocation that share many of the same patient-specific risk factors were notably not significantly different across groups. Further evaluation is required to determine root causes and possible interventions for minimizing this complication.

This study has several limitations. First, the data were collected using a large statewide registry, and therefore the findings may have limited generalizability outside of Michigan. Although specifically abstracted by trained nurse analysts, the granularity of information may be decreased. The specific reasons for postoperative ER visits and readmissions were not captured, though prior studies report that 60% of the 90-day ER visits and 75% of 90-day readmissions are related to the TJA rather than an unrelated condition [27,32]. Moreover, value-based performance metrics and bundled payment systems often associate all 90-day postoperative ER visits and readmissions with the index surgery regardless of their cause [[33], [34], [35]]. Patient-reported outcomes were another outcome not captured in our registry. While many inpatient facilities have been collecting patient-reported outcomes, this is new to ASC setting, and the data are not yet robust enough to report upon. Second, an inherent selection bias exists in the ASC population with older, more medically complex, and government payer patients receiving primary TJA in the HOPDs and hospitals, as noted in prior studies [5,[8], [9], [10], [11], [12]]. The significant demographic differences preclude direct comparison of outcomes. The objective of the present study was to validate previously described demographic and outcome trends across different surgery settings using a state-wide, abstracted registry in the years following the elective shutdown for the COVID-19 pandemic. We did not attempt to utilize matching or logistic regression analyses to directly assess outcomes specific to each setting, as this would risk excluding the sickest patient populations.

## Conclusions

One year after the COVID-19 pandemic, primary TJA at ASCs and HOPDs continues to grow in Michigan. Despite a lack of definitive appropriateness criteria, surgeons have been selecting patients for alternate sites of care (ASC and HOPD) with successful outcomes. Primary TJA performed at ASCs had the lowest readmission rates and ER visits compared to HOPDs and hospitals with essentially similar rates of PJI, periprosthetic hip fracture, and prosthetic hip dislocation. Hospitals continue to manage the most complex patients, with nearly one fifth of the hospital cohort requiring more than 1 night’s hospital admission after their surgery. Predictably, this cohort had the highest rates of requiring postdischarge hospital care. However, given that almost one third of the hospital patients were discharged on the day of surgery and the continued success of TJA at ASCs and HOPDs, we predict continued evolution of patient selection and postoperative care.

## Conflicts of interest

E. Dailey is an editorial board member of Arthroplasty Today. D. C. Markel receives royalties from Smith & Nephew; is a paid consultant for Smith & Nephew and Stryker; has stock options in Arboretum Ventures, HOPCo, and Plymouth Capital; receives research support from Ascension Providence Hospital and Stryker; is an editorial board member of Arthroplasty Today and Journal of Arthroplasty; and is a board or committee member of Michigan Arthroplasty Registry Collaborative Quality Initiative and Michigan Orthopaedic Society. All other authors declare no potential conflicts of interest.

For full disclosure statements refer to https://doi.org/10.1016/j.artd.2025.101659.

## CRediT authorship contribution statement

**Simarjeet Puri:** Writing – review & editing, Writing – original draft, Project administration, Data curation. **Martin Weaver:** Writing – review & editing, Writing – original draft, Project administration, Conceptualization. **Lisheng Chen:** Software, Resources, Methodology, Formal analysis, Data curation, Conceptualization. **Tae Kim:** Supervision, Software, Methodology, Investigation, Formal analysis, Data curation, Conceptualization. **Elizabeth Dailey:** Writing – review & editing, Writing – original draft, Project administration, Conceptualization. **David C. Markel:** Writing – review & editing, Writing – original draft, Investigation, Conceptualization.

## Appendix A Supplementary data

Conflict of Interest Statement for Chen

Conflict of Interest Statement for Dailey

Conflict of Interest Statement for Kim

Conflict of Interest Statement for Markel

Conflict of Interest Statement for Puri

Conflict of Interest Statement for Weaver
