# Supplementary material for: Assessment of 90-Day Outcomes Following Total Joint Arthroplasty in Ambulatory Surgery Centers, Hospital Outpatient Departments, and Hospitals: A Michigan Arthroplasty Registry Collaborative Quality Initiative Analysis
Source: Arthroplast Today. 2025 Mar 8;32:101659. doi: 10.1016/j.artd.2025.101659 (PMC11930425; doi:10.1016/j.artd.2025.101659)
Supplement: Conflict of Interest Statement for Markel [file mmc4.docx]

# INDIVIDUAL CONFLICT OF INTEREST STATEMENT

***American Association of Hip and Knee Surgeons***

(Adopted from the American Academy of Orthopaedic Surgeons disclosure statement)

The following form **must be filled out completely and submitted by each author (example, 6 authors, 6 forms).**

**All items require a response. If there is no relevant disclosure for a given item, enter "*None*.”**

**Assessment of 90-day outcomes following total joint arthroplasty in ambulatory surgery centers, hospital outpatient departments, and hospitals: A Michigan Arthroplasty Registry Collaborative Quality Initiative Analysis**

**Manuscript Title**

1. Royalties from a company or supplier (The following conflicts were disclosed)

**Smith & Nephew**

2. Speakers bureau/paid presentations for a company or supplier (The following conflicts were disclosed)

**none**

3A. Paid employee for a company or supplier (The following conflicts were disclosed)

**none**

3B. Paid consultant for a company or supplier (The following conflicts were disclosed)

**Smith & Nephew, Stryker**

3C. Unpaid consultants for a company or supplier (The following conflicts were disclosed)

**none**

4. Stock or stock options in a company or supplier (The following conflicts were disclosed)

**Arboretum Ventures, HOPCo, Plymouth Capital**

5. Research support from a company or supplier as a Principal Investigator (The following conflicts were disclosed)

**Ascension Providence Hospital, Stryker**

6. Other financial or material support from a company or supplier (The following conflicts were disclosed)

**none**

7. Royalties, financial or material support from publishers (The following conflicts were disclosed)

**none**

8. Medical/Orthopaedic publications editorial/governing board (The following conflicts were disclosed)

**Arthroplasty Today, Journal of Arthroplasty**

9. Board member/committee appointments for a society (The following conflicts were disclosed)

**Michigan Arthroplasty Registry Collaborative Quality Initiative, Michigan Orthopedic Society**

**Each author must sign AND print or type his/her name, date and submit a separate form**



In addition, one BLINDED Conflict of Interest form (no author names used) should be submitted per manuscript with all author disclosures.

David C. Markel 6/10/2024


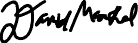


Author Name (Print or Type) Author Signature Date
